# Cytokine Profiles during Invasive Nontyphoidal Salmonella Disease Predict Outcome in African Children

**DOI:** 10.1128/CVI.00128-16

**Published:** 2016-07-05

**Authors:** James J. Gilchrist, Jennifer N. Heath, Chisomo L. Msefula, Esther N. Gondwe, Vivek Naranbhai, Wilson Mandala, Jenny M. MacLennan, Elizabeth M. Molyneux, Stephen M. Graham, Mark T. Drayson, Malcolm E. Molyneux, Calman A. MacLennan

**Affiliations:** a Wellcome Trust Centre for Human Genetics, University of Oxford, Oxford, United Kingdom; b Department of Paediatrics, University of Oxford, Oxford, United Kingdom; c School of Immunity and Infection, College of Medicine and Dental Sciences, University of Birmingham, Birmingham, United Kingdom; d Malawi-Liverpool-Wellcome Trust Clinical Research Programme, College of Medicine, University of Malawi, Blantyre, Malawi; e Department of Microbiology, College of Medicine, University of Malawi, Blantyre, Malawi; f Liverpool School of Tropical Medicine, Pembroke Place, Liverpool, United Kingdom; g Department of Zoology, University of Oxford, Oxford, United Kingdom; h Department of Paediatrics, College of Medicine, University of Malawi, Blantyre, Malawi; i Centre for International Child Health, University of Melbourne and Murdoch Children's Research Institute, Royal Children's Hospital, Melbourne, Australia; j Department of Medicine, College of Medicine, University of Malawi, Blantyre, Malawi; k Jenner Institute, Nuffield Department of Medicine, University of Oxford, Oxford, United Kingdom; l Wellcome Trust Sanger Institute, Wellcome Trust Genome Campus, Hinxton, Cambridge, United Kingdom; University of Florida

## Abstract

Nontyphoidal Salmonella is a leading cause of sepsis in African children. Cytokine responses are central to the pathophysiology of sepsis and predict sepsis outcome in other settings. In this study, we investigated cytokine responses to invasive nontyphoidal Salmonella (iNTS) disease in Malawian children. We determined serum concentrations of 48 cytokines with multiplexed immunoassays in Malawian children during acute iNTS disease (*n* = 111) and in convalescence (*n* = 77). Principal component analysis and logistic regression were used to identify cytokine signatures of acute iNTS disease. We further investigated whether these responses are altered by HIV coinfection or severe malnutrition and whether cytokine responses predict inpatient mortality. Cytokine changes in acute iNTS disease were associated with two distinct cytokine signatures. The first is characterized by increased concentrations of mediators known to be associated with macrophage function, and the second is characterized by raised pro- and anti-inflammatory cytokines typical of responses reported in sepsis secondary to diverse pathogens. These cytokine responses were largely unaltered by either severe malnutrition or HIV coinfection. Children with fatal disease had a distinctive cytokine profile, characterized by raised mediators known to be associated with neutrophil function. In conclusion, cytokine responses to acute iNTS infection in Malawian children are reflective of both the cytokine storm typical of sepsis secondary to diverse pathogens and the intramacrophage replicative niche of NTS. The cytokine profile predictive of fatal disease supports a key role of neutrophils in the pathogenesis of NTS sepsis.

## INTRODUCTION

In sub-Saharan Africa, nontyphoidal Salmonella (NTS) causes invasive and frequently fatal disease in young children and HIV-infected adults (1). Invasive NTS (iNTS) disease typically manifests as bacteremia, presenting with a syndrome of febrile illness commonly complicated by sepsis (1, 2). In 2010, iNTS disease was estimated to have caused 3.4 million episodes of illness globally, resulting in nearly 700,000 deaths, the large majority of which occurred in Africa (3). The high burden of morbidity and mortality associated with iNTS disease in Africa reflects inadequate control strategies. There is no NTS vaccine available, and expanding multidrug resistance renders many commonly available antibiotics ineffective (4). The delivery of new interventions to control iNTS disease in Africa will be facilitated by an improved understanding of the biology of these infections.

The host immune response to sepsis is characterized by pro- and anti-inflammatory changes, reflected in circulating peripheral cytokine levels (5). This diverse host immune response is central to the pathophysiology of bacteremia and sepsis, and interindividual differences in circulating cytokine profiles are associated with sepsis outcome (6, 7). Increased concentrations of both proinflammatory (e.g., tumor necrosis factor [TNF] [8], interleukin-6 [IL-6] [9], and IL-8 [10]) and anti-inflammatory (e.g., IL-1Ra [11], IL-10, and high IL-10/TNF ratios [6, 7]) cytokines in patients with sepsis have been previously demonstrated to predict mortality. While much of the characteristic sepsis-associated cytokine response is seen regardless of its etiology, there is evidence for pathogen specificity in cytokine responses during sepsis despite causing clinically indistinguishable syndromes (12, 13). In this study, we used multiplexed immunoassays to describe cytokine profiles associated with acute NTS bacteremia in Malawian children. We investigated whether these profiles are modified by common comorbidities (HIV infection and malnutrition) associated with iNTS disease in African children and whether distinct cytokine profiles in acute infection are predictive of mortality.

## MATERIALS AND METHODS

### Study site and participants.

Queen Elizabeth Central Hospital (QECH), Blantyre, is the largest government hospital in Malawi. Since the implementation of clinical bacterial culture services in 1998, all febrile children without malaria parasitemia and all children with suspected sepsis regardless of malaria parasitemia are investigated with blood culture on admission to QECH. In 2006, 249 children were admitted to QECH with NTS bacteremia. Children under 16 years of age were eligible for recruitment into the study approximately 24 h after admission, following isolation of NTS by blood culture. Bacterial culture from blood was performed with the BacT/Alert 3D system (bioMérieux), and identification of Salmonella and serotyping was done using API 20E kits (bioMérieux) and agglutinating antisera (Difco Laboratories). Plasmodium falciparum parasitemia was tested by thick and thin blood films. HIV status was determined using Determine (Abbot Laboratories) and UniGold (Trinity Biotech) rapid tests, and HIV infection was confirmed in children less than 18 months of age by PCR. Children with weight for height (children <60 months) or body mass index for age (children >60 months) Z scores greater than 3 standard deviations below WHO median values or children with bilateral pedal edema (kwashiorkor) were classified as severely malnourished. Full blood counts were performed using an HMX (Becton Coulter).

Of 249 children with NTS bacteremia admitted to QECH in 2006, 111 (45%) were recruited to the study and a blood sample was obtained during their acute illness. Of the remaining 138 children, 25 died prior to recruitment and 14 were discharged or left the hospital prior to their blood culture results becoming available. For the other 99 children, consent for blood sampling was not obtained (*n* = 38), blood samples were not collected for clinical reasons following recruitment (e.g., anemia requiring transfusion; *n* = 42), or children could not be located (*n* = 19). One hundred one of 111 children had Salmonella enterica serovar Typhimurium and 8 had *S*. Enteritidis isolated from their blood. Two Salmonella isolates were untypeable with the available antisera. The median age was 16 months (range, 4 to 72 months). Forty-eight of 109 children were HIV infected (44%), of whom 2 were receiving antiretroviral therapy (ART) at presentation. Twenty-nine of 103 children were severely malnourished (28%). Only 6 children had malaria parasitemia; therefore, comparisons were not made for children according to malaria status. Fourteen of 111 children (12%) died following recruitment. This case fatality is lower than that seen in unselected cases of acute iNTS in Malawian children in 2006 (20%), and it reflects the high number of children with NTS bacteremia that die following admission but prior to their blood culture indicating a diagnosis of Gram-negative bacteremia. Sera for cytokine quantification were collected during acute iNTS disease (median of 1 day following collection of blood culture; range, 0 to 5 days) in all 111 children and in convalescence (median interval, 43 days; range, 31 to 67 days) in 77. No children were recorded as having died during the period following discharge. As part of routine clinical care, a peripheral blood leukocyte count was obtained in 79 children during acute disease, with a white blood cell differential count being performed in 52. Study participant characteristics are presented in Table 1.

**TABLE 1:** Characteristics and comorbidities of study participants^*a*^

Parameter	Value by sample type	
	Acute (*n* = 111)	Convalescent (*n* = 77)
Demographics, comorbidity, and outcome		
Age (mo; median, range)	16, 4–72	18, 4–72
Female (no., %)	52/111, 47	35/77, 45
HIV infected (no., %)	48/109, 44	32/77, 42
Severe malnutrition (no., %)	29/103, 28	17/72, 24
Malaria (no., %)	6/108, 6	5/75, 7
Inpatient mortality (no., %)	14/111, 12	
Clinical status at admission^*b*^ (no., %)		
Febrile	88/104, 85	
Tachycardia	19/31, 61	
Tachypnea	25/36, 69	
BCS < 5	9/104, 9	
Respiratory distress (no., %)	30/81, 37	
Full blood count^*c*^		
Hemoglobin (g/dl; median, range)	8.0, 3.9–14.4	
Leukocytes (×10^9^/liter; median, range)	8.0, 1.3–37.8	
Neutrophils (×10^9^/liter; median, range)	2.7, 0.3–14.2	
Lymphocytes (×10^9^/liter; median, range)	3.8, 0.3–10.3	

a Participant characteristics for both acute- and convalescent-phase samples represent status at initial presentation with acute invasive nontyphoidal Salmonella disease.

b Tachycardia and tachypnea were defined as rates greater than the 90th percentile for age, and respiratory distress was defined as the presence of tracheal tug, intercostal or subcostal recession, head bobbing, or nasal flaring. BCS, Blantyre coma score.

c Hemoglobin concentrations and white blood cell counts were determined in 93 and 79 participants, respectively, with acute disease. White blood cell differential counts were reported in 52 participants with acute disease.

### Serum cytokine quantification.

Sera were separated from clotted blood within 2 h of venesection and stored at −80°C. Serum concentrations of 48 cytokines (see Table S1 in the supplemental material) were assayed using Bio-Plex Pro 21- and 27-plex human cytokine, chemokine, and growth factor fluorescent bead-based assays (Bio-Rad Laboratories). Paired acute- and convalescent-phase samples were assayed together. In brief, antibody-coated fluorescent microspheres were incubated with manufacturer-supplied cytokine standards and study sera, washed, and incubated with detection antibody. Following washing, the microspheres were incubated with streptavidin-conjugated phycoerythrin before data acquisition on a Luminex-100 instrument (Bio-Rad Laboratories) using Bio-Plex Manager 4.1.1 software (Bio-Rad). Cytokine measurements below the detection limit of the assay were assigned values of the lower detection limit for each cytokine and were included in the analysis. At least one multiplexed assay failed in the case of five samples (3 acute, 2 convalescent), and these were excluded from analysis.

### Statistical analysis.

Paired acute- and convalescent-phase serum cytokine concentrations were compared with paired Wilcoxon signed-rank tests. Acute-phase serum cytokine concentrations in children with and without HIV infection and severe malnutrition, and in children who survived and children who died, were compared using Mann-Whitney U tests. *P* values were adjusted for multiple comparisons with Holm's corrections. Heat maps were generated using Gene Cluster v3.0 and visualized in TreeView v1.60 (Michael Eisen, Stanford University). Following log transformation of the cytokine concentrations, principal component analysis (14) was performed using cytokines differing between groups of interest (*P*_adjusted_ < 0.05). Principal components (PC) were extracted with eigenvalues of >1.0 and with at least three significantly associated cytokines (factor loadings of >0.4 or <−0.4) and were obliquely rotated. The extracted principal components then were included in multivariable logistic regression models. Leukocyte counts were normalized prior to analysis with appropriate transformations and correlated with independent variables with linear regression. All statistical analyses were performed in R.

### Ethics approval.

Ethical approval was granted by the College of Medicine Research and Ethics Committee, University of Malawi. Informed written consent was obtained from the parents or guardians of each child.

## RESULTS

### Cytokine responses in acute iNTS disease.

**FIG 1:**
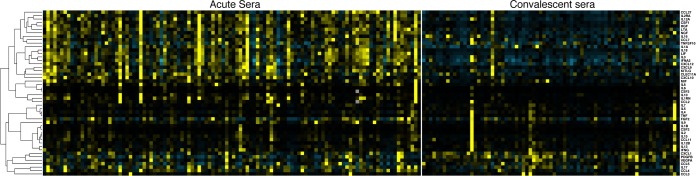
Serum cytokine changes in acute iNTS disease and in convalescence. Shown is a heat map of 48 serum cytokine concentrations in acute iNTS disease (left; *n* = 108) and in convalescence (right; *n* = 75). Yellow color change represents increased cytokine concentration relative to the median for all samples, and blue color change represents a decrease. Cytokines are ordered by unsupervised hierarchical clustering, with between-cytokine distances represented by the dendrogram on the left side of the heat map.

**FIG 2:**
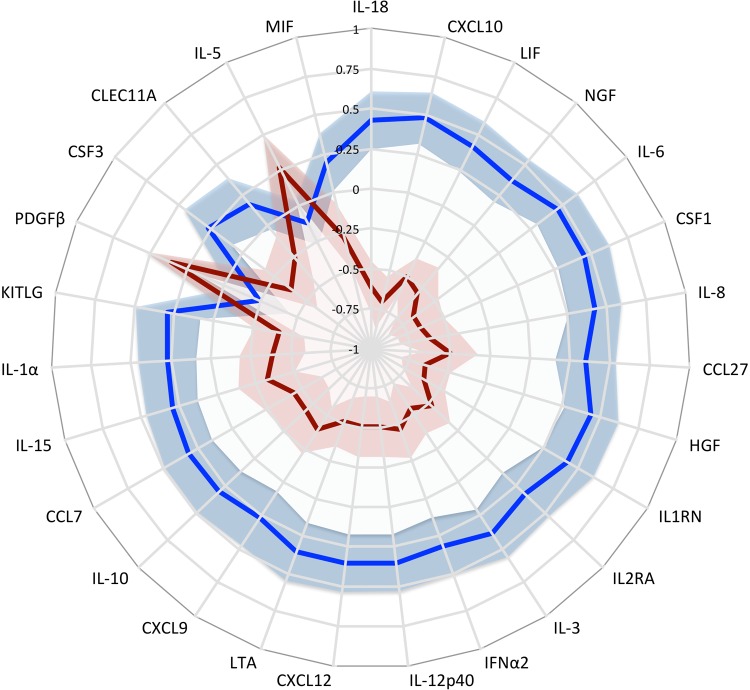
Serum cytokine changes in acute iNTS disease and in convalescence. Shown is a radar plot of the serum concentrations of cytokines (*n* = 27) significantly differentiated between acute iNTS disease (blue; *n* = 108) and convalescence (red; *n* = 75). Lower serum cytokine concentrations plot toward the center of the graph, and shaded areas represent 95% confidence intervals. Cytokine concentrations are normalized and standardized with rank-based inverse normal transformations.

Serum cytokine concentrations during acute iNTS disease have a distinct profile compared with that seen in convalescence (Fig. 1). Following correction for multiple association testing, serum concentrations of 27 of 48 measured cytokines are significantly altered (*P*_adjusted_ < 0.05) during acute iNTS disease compared with those during convalescence (Table 2). Of the cytokines previously associated with acute disease, the levels of 25 of 27 were significantly increased in acute disease (Fig. 2).

**TABLE 2:** Serum cytokine concentrations during acute iNTS disease and in convalescence^*a*^

Cytokine	Median (IQR) cytokine concn (pg/ml)		*P* value	
	Acute	Convalescent	Unadjusted	Adjusted
IL-18	1,232 (627–2,122)	345 (195–510)	1.6 × 10^−12^	7.7 × 10^−11^
CXCL10	17,922 (9,512–30,037)	5,093 (3,291–7,393)	1.2 × 10^−11^	5.7 × 10^−10^
NGF	10.1 (7.2–14.3)	5.7 (3.7–7.2)	1.3 × 10^−10^	5.9 × 10^−9^
CCL27	1,096 (845–1,538)	699 (552–877)	1.9 × 10^−10^	8.4 × 10^−9^
LIF	33.7 (9.0–61.9)	1.9 (1.9–12.6)	2.7 × 10^−10^	1.2 × 10^−8^
CSF1	42.1 (28.1–73.9)	15.7 (10.8–19.6)	2.9 × 10^−10^	1.2 × 10^−8^
HGF	2,063 (1,064–2,820)	704 (562–1,014)	3.8 × 10^−10^	1.6 × 10^−8^
IL-6	43.6 (22.0–105.4)	8.8 (7.1–16.4)	9.4 × 10^−10^	3.9 × 10^−8^
IL-2Ra	972 (568–1,664)	467 (264–732)	1.2 × 10^−9^	4.8 × 10^−8^
IL-8	36.3 (26.4–66.3)	18.4 (13.5–27.0)	1.4 × 10^−9^	5.4 × 10^−8^
IL-1Ra	782 (287–1,844)	187 (126–285)	4.4 × 10^−9^	1.7 × 10^−7^
IFN-α2	164.7 (126.5–206.2)	113.1 (92.3–137.2)	6.1 × 10^−9^	2.3 × 10^−7^
IL-3	344.2 (178.8–467.6)	125.9 (74.3–183.7)	7.1 × 10^−9^	2.5 × 10^−7^
IL-12p40	666 (463–1,007)	322 (230–478)	2.2 × 10^−8^	7.9 × 10^−7^
CXCL12	256.2 (201.3–328.1)	146.2 (114.7–194.6)	3.8 × 10^−8^	1.3 × 10^−6^
LTA	6.2 (3.8–8.1)	3.0 (2.2–3.9)	2.1 × 10^−7^	6.8 × 10^−6^
CXCL9	12,444 (6,836–25,961)	6,116 (3,511–9,494)	5.0 × 10^−7^	1.6 × 10^−5^
KITLG	197.7 (123.1–262.7)	118.9 (98.1–147.4)	2.6 × 10^−6^	8.0 × 10^−5^
CCL7	47.1 (28.6–74.2)	24.1 (14.6–37.4)	2.9 × 10^−6^	8.6 × 10^−5^
IL-10	14.7 (7.5–34.7)	5.9 (4.4–10.5)	3.2 × 10^−6^	9.3 × 10^−5^
IL-1α	2.6 (1.5–3.6)	1.4 (0.5–2.3)	4.5 × 10^−6^	1.3 × 10^−4^
IL-15	14.7 (2.2–24.8)	1.3 (1.3–9.3)	1.0 × 10^−5^	2.8 × 10^−4^
PDGFβ	3,954 (1,867–7,985)	7,433 (4,827–11,246)	1.7 × 10^−5^	4.5 × 10^−4^
CLEC11a	61,008 (41,516–105,760)	42,730 (33,827–59,077)	3.5 × 10^−5^	8.7 × 10^−4^
CSF3	43.1 (23.7–67.1)	22.9 (13.1–36.0)	4.2 × 10^−5^	1.0 × 10^−3^
IL-5	0.8 (0.1–1.9)	1.7 (0.1–2.9)	2.1 × 10^−4^	4.8 × 10^−3^
MIF	742 (476–1,226)	558 (333–764)	8.3 × 10^−4^	0.02

a Data from 146 participant samples (73 matched acute- and convalescent-phase sample pairs) are included in the analysis. Significance testing was performed with paired Wilcoxon signed-rank tests, with *P* values adjusted for multiple comparisons with Holm step-down corrections. Serum cytokine concentrations significantly altered in acute disease compared to those in convalescence (*P*_adjusted_ < 0.05; *n* = 27) are displayed. IQR, interquartile range.

To better delineate the biological processes underlying these cytokine changes, we included the 27 cytokines in a principal component analysis of serum cytokine concentration changes in acute iNTS disease. Two principal components (PC) with eigenvalues greater than 1 were extracted which cumulatively explain 59% of the variance in cytokine responses (Table 3). Using conditional logistic regression, in 73 matched acute- and convalescent-phase sample pairs, PC1_iNTS_ (odds ratio [OR], 10.36; 95% confidence interval [CI], 3.69 to 29.06; *P* = 8.8 × 10^−6^) and PC2_iNTS_ (OR, 7.26; 95% CI, 3.02 to 17.45; *P* = 9.29 × 10^−6^) are significantly associated with acute iNTS disease. The inspection of the factor loadings of each principal component (Table 3) suggests that each PC represents a qualitatively different component of the host immune response to iNTS disease. PC1_iNTS_ is characterized by increased concentrations of mediators and markers known to be associated with macrophage function, with raised levels of cytokines impacting their differentiation (colony-stimulating factor 1 [CSF1] and IL-3 [15]), migration (NGF, CXCL12 [16], and CCL7 [17]), activation (IL-12, IL-18 [18], and CXCL9 [19]), and survival (alpha interferon [IFN-α] [20]). PC1_iNTS_ is also characterized in part by raised circulating markers of T cell migration (CCL27 [21]) and activation (IL-2Rα [22]). PC2_iNTS_ is characterized by a cytokine profile typically described in patients with sepsis, with concomitantly raised proinflammatory (IL-6, IL-8, CXCL10, and IL-15) and anti-inflammatory (IL-10 and IL-1Ra) cytokines (23).

**TABLE 3:** Principal component analysis of serum cytokine concentrations differentiating acute iNTS disease and convalescence^*a*^

Cytokine	Analysis result^*b*^	
	PC1_iNTS_	PC2_iNTS_
LTA	**0.94**	−0.15
CSF1	**0.92**	−0.03
NGF	**0.88**	0
IL-3	**0.85**	0.1
CCL7	**0.83**	−0.03
LIF	**0.83**	−0.06
IFNα2	**0.81**	0
IL-2Ra	**0.81**	0.07
KITLG	**0.81**	0.1
IL-12p40	**0.78**	0.16
IL-18	**0.77**	0.07
CXCL9	**0.72**	0.1
CCL27	**0.65**	0.06
HGF	**0.54**	**0.43**
CXCL12	**0.5**	0.33
IL-1Ra	0.03	**0.88**
IL-6	0.06	**0.85**
CSF3	−0.05	**0.77**
IL-10	0.13	**0.72**
CXCL10	0.07	**0.7**
IL-15	−0.02	**0.68**
IL-8	0.24	**0.6**
CLEC11A	0.11	**0.5**
MIF	0.23	0.34
PDGFβ	−0.12	−0.3
IL-5	0.04	0.01
IL-1α	0.18	0.33
Eigenvalue	10.26	5.62
Proportion variance	0.38	0.21

a Measurements of 27 cytokines in 146 participant samples (73 matched acute- and convalescent-phase sample pairs) are included in the analysis.

b For each PC, the factor loading of each cytokine included in the analysis is displayed. Factor loadings represent the correlation between each cytokine and a principal component, varying between −1 and +1. The larger the absolute value of a given factor loading, the greater importance of that cytokine to the principal component. We define influential cytokines for a given principal component as having a factor loading of >0.4 or <−0.4. Cytokines with loadings in excess of ±0.4 account for at least 16% of the variance in the principal component. Influential cytokines for each principal component are displayed in boldface.

### iNTS disease cytokine responses in HIV-coinfected and malnourished children.

**FIG 3:**
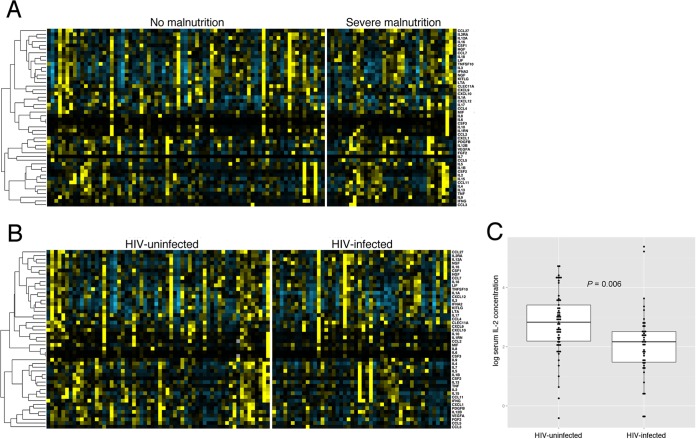
Serum cytokine responses to acute iNTS disease in African children with HIV infection and malnutrition. (A) Heat map of 48 serum cytokine concentrations during acute iNTS disease in children with (right; *n* = 27) and without (left; *n* = 73) severe malnutrition. (B) Heat map of 48 serum cytokine concentrations during acute iNTS disease in children with (right; *n* = 47) and without (left; *n* = 59) HIV coinfection. Yellow color change represents increased cytokine concentration relative to the median for all samples, and blue color change represents a decrease. Cytokines are ordered by unsupervised hierarchical clustering, with between-cytokine distances represented by the dendrogram on the left side of the heat map. (C) Serum IL-2 concentration (log transformed) in acute iNTS disease in uninfected (left; *n* = 47) and HIV-infected (right; *n* = 59) children.

Examination of the cytokine profiles in acute iNTS disease does not demonstrate clear differences between HIV-infected and uninfected children or between children with and without severe malnutrition (Fig. 3). Indeed, following correction for multiple comparisons, no cytokine concentration was significantly affected by nutritional status (data not shown). Only IL-2 was found to vary with HIV status, with the IL-2 concentration in acute iNTS disease being significantly decreased in HIV-infected children (median IL-2_HIV infected_ level, 7.94 pg/ml; IL-2_HIV uninfected_ level, 16.93 pg/ml; *P*_adjusted_ = 0.006) (Fig. 3C).

### Cytokine profiles in fatal iNTS disease.

**FIG 4:**
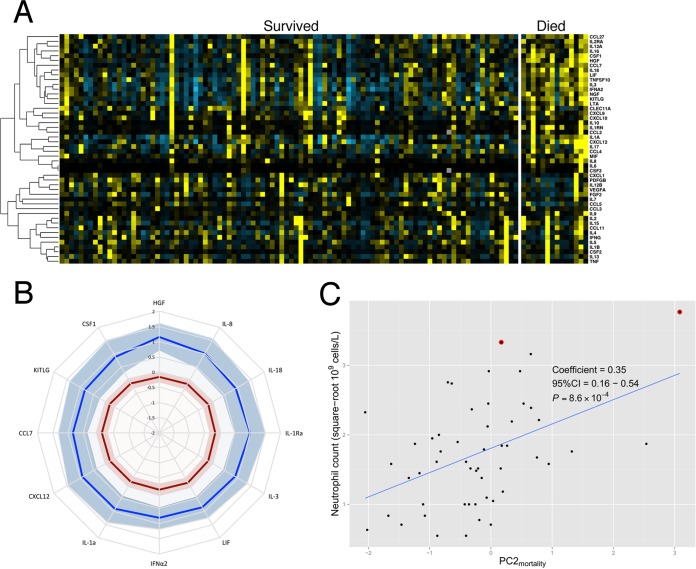
Serum cytokine responses to acute iNTS disease in African children with fatal and nonfatal disease. (A) Heat map of 48 serum cytokine concentrations during acute iNTS disease in children with nonfatal (left; *n* = 94) and fatal (right; *n* = 14) disease. Yellow color change represents increased cytokine concentration relative to the median for all samples, and blue color change represents a decrease. Cytokines are ordered by unsupervised hierarchical clustering, with between-cytokine distances represented by the dendrogram on the left side of the heat map. (B) Radar plot of the serum concentrations of cytokines (*n* = 12) in acute iNTS disease significantly differentiated between fatal (blue; *n* = 14) and nonfatal (red; *n* = 94) disease. Lower serum cytokine concentrations plot toward the center of the graph, and shaded areas represent 95% confidence intervals. Cytokine concentrations are normalized and standardized with rank-based inverse normal transformations. (C) Scatter plot of peripheral blood neutrophil counts and PC2_mortality_ scores in Malawian children (*n* = 52) with acute iNTS disease. The linear regression line is plotted (blue). Two children with neutrophil count data subsequently died (data points highlighted with red circles).

A comparison of the cytokine profiles during acute iNTS disease between survivors and children who died in the hospital following recruitment demonstrates that fatal iNTS disease has a distinctive cytokine profile (Fig. 4). Serum concentrations of 12 of 48 cytokines during acute iNTS disease were significantly different in nonsurvivors than in survivors (see Table S2 in the supplemental material), with all differentially expressed cytokines being increased in children who subsequently died (Fig. 4). We included these 12 cytokines in a principal component analysis to identify any underlying structure in this group of cytokines, which we hypothesized would be representative of distinct immunological processes. Analysis revealed two principal components with eigenvalues greater than 1 (Table 4), which cumulatively explain 74% of the variance in cytokine responses associated with mortality. To test if either of these principal components independently predicts mortality, we fitted a multivariable logistic regression model of mortality including both principal components as covariates. In this model, PC2_mortality_ (OR, 4.22; 95% CI, 1.63 to 12.24; *P* = 0.005; see Table S3), but not PC1_mortality_ (OR, 1.64; 95% CI, 0.65 to 7.90; *P* = 0.448), is associated with iNTS disease mortality in Malawian children. To further investigate this observation, we fitted a second multivariable logistic regression model of mortality including PC2_mortality_, HIV infection, and malnutrition as covariates, demonstrating that the observed association of PC2_mortality_ (OR, 5.11; 95% CI, 2.29 to 14.18; *P* = 3.6 × 10^−4^) with mortality is independent of HIV infection and severe malnutrition (see Table S4). Inspection of the factor loadings (Table 4) demonstrates that PC2 is characterized by increased IL-8 and HGF and perturbed IL-1 signaling with increased IL-1Ra and IL-1α levels, all factors associated with neutrophil function. The coregulation of this group of cytokines suggests that PC2_mortality_ reflects the migration and activation of neutrophils. Both IL-8 and HGF direct the recruitment and activation of neutrophils at sites of inflammation, and signaling via both cytokines is induced by IL-1 signaling (24, 25). To further understand the role of this group of coregulated cytokines in neutrophil function, we tested for an association with the PC2_mortality_ and peripheral blood neutrophil counts during acute disease. Square-root-transformed neutrophil counts were significantly associated by linear regression (*P* = 8.6 × 10^−4^) (Fig. 4) with PC2_mortality_ during acute disease. This association appears to be specific to circulating neutrophil numbers, with no significant associations demonstrated between PC2_mortality_ and log-transformed circulating total leukocyte (*P* = 0.51, *n* = 79), monocyte (*P* = 0.39, *n* = 52), or lymphocyte (*P* = 0.39, *n* = 52) numbers. In a multiple-regression model, PC2_mortality_ is significantly associated with neutrophil count independent of age, sex, malnutrition, and HIV infection (*P* = 0.04) (see Table S5).

**TABLE 4:** Principal component analysis of serum cytokine concentrations in acute iNTS disease differentiating fatal and nonfatal diseases^*a*^

Cytokine	Analysis result^*b*^	
	PC1_mortality_	PC2_mortality_
LIF	**0.97**	−0.16
IL-18	**0.90**	−0.03
IL-3	**0.90**	0.08
IFNα2	**0.89**	0.00
CCL7	**0.84**	0.03
KITLG	**0.79**	0.12
CSF1	**0.79**	−0.05
CXCL12	**0.61**	0.35
IL-1α	**0.45**	**0.40**
IL-1Ra	−0.01	**0.94**
IL-8	−0.01	**0.85**
HGF	**0.41**	**0.53**
Eigenvalue	6.37	2.56
Proportion variance	0.53	0.21

a Measurements of 12 cytokines in 108 participant samples are included in the analysis.

b For each PC, the factor loading of each cytokine included in the analysis is displayed. Factor loadings represent the correlation between each cytokine and a principal component, varying between −1 and +1. The larger the absolute value of a given factor loading, the greater importance of that cytokine to the principal component. We define influential cytokines for a given principal component as having a factor loading of >0.4 or <−0.4. Cytokines with loadings in excess of ±0.4 account for at least 16% of the variance in the principal component. Influential cytokines for each principal component are displayed in boldface.

## DISCUSSION

In this study, we describe the cytokine profile associated with acute iNTS disease in African children. The response is composed of two biologically distinct principal components. The first group of coregulated cytokines is consistent with a role for macrophage differentiation, migration, and function during acute NTS bacteremia. The association of acute iNTS disease with a group of coregulated cytokines known to be involved with macrophage function is consistent with our understanding of iNTS pathogenesis. NTS is a facultative intracellular pathogen, and the control of intracellular NTS infection within macrophages is central to host immunity to iNTS in both animal models and human disease (26). In human disease, this is most clearly illustrated by Mendelian susceptibility to mycobacterial disease (MSMD). MSMD is a genetically heterogeneous collection of primary immunodeficiencies with disseminated NTS disease reported in half of cases (27) and in all of which intramacrophage control of NTS is compromised (26).

The second group of coregulated cytokines, associated with acute iNTS disease, appears to represent a less Salmonella-specific immune response, with increased pro- and anti-inflammatory cytokines typical of the dysregulated cytokine storm described for patients with sepsis regardless of its etiology (5). The demonstration of concomitantly increased pro- and anti-inflammatory cytokines at admission with acute iNTS disease is in keeping with our current understanding of the pathophysiology of sepsis, in which both responses are seen very early in the course of disease (23).

It is striking that the cytokine response to acute iNTS disease in Malawian children was largely unaffected by the presence of either severe malnutrition or HIV infection in this study. No cytokine is significantly perturbed (following correction for multiple association testing) during acute iNTS disease by the presence of severe malnutrition. Only IL-2 is significantly increased in HIV-infected children with iNTS during acute infection, despite only 2 of 48 HIV-infected children receiving ART prior to admission. This observation may reflect the study's power to detect differentially expressed cytokines following correction for multiple association testing, but it is in keeping with previously published data which report minimal changes in the cytokine response to sepsis in HIV-infected and -uninfected individuals (28). This is noteworthy, as HIV infection increases the risk of mortality secondary to sepsis (29), and well-characterized cytokine perturbations are described during both acute and chronic HIV infection (30). In contrast, HIV-infected Malawian children with invasive pneumococcal disease (IPD) have plasma concentrations of CXCL8 and CCL2 that are increased compared to those of HIV-uninfected children with IPD (31). This difference is likely to be largely attributable to differences in the invasive bacterial disease, as the large majority of the children included in the IPD study had pneumococcal meningitis rather than bacteremia.

A distinctive cytokine profile predicts case fatality in children with acute NTS bacteremia. That cytokine profile is characterized by increased HGF and IL-1 (IL-1α and IL-1Ra) signaling and increased levels of IL-8. IL-1 signaling molecules include two IL-1 receptor agonists (IL-1α and IL-1β) and one IL-1 receptor antagonist, IL-1Ra, which together determine the level of functional IL-1 signaling via the type 1 IL-1 receptor (32). In contrast to IL-1β, IL-1α is constitutively expressed in nonhematopoietic cells (33). This constitutive expression facilitates a rapid inflammatory response to necrosis in a wide variety of tissues (33). In response to tissue necrosis, IL-1α signaling results in IL-8 production by endothelial cells (34), which recruits neutrophils to sites of tissue damage (24). IL-1Ra, a competitive inhibitor of IL-1 signaling, is produced in parallel to IL-1 during the acute inflammatory response (35) and antagonizes functional IL-1 signaling, including IL-8 production (34).

HGF signaling via its cognate receptor tyrosine kinase, MET, supports the proliferation, migration, and morphogenesis of epithelial cells (36). More recently, however, HGF-MET signaling has been demonstrated to mediate neutrophil transendothelial migration at sites of inflammation (25). In addition to its stimulation of IL-8 production by endothelial cells, endothelial activation by IL-1α increases the expression of MET by neutrophils (25). Taken together, fatal iNTS disease is characterized by a cytokine profile characteristic of neutrophil recruitment to sites of tissue damage. The observation that this group of cytokines is also associated with circulating neutrophil numbers suggests that these cytokines support not only tissue recruitment of neutrophils but also emergency granulopoiesis during acute iNTS disease. This effect may be indirect, and cytokines classically implicated in emergency granulopoiesis (37) (e.g., CSF2, CSF3, and IL-6) are not associated with the cytokine signature predictive of mortality. However, there is evidence for the role of IL-1 signaling in reactive granulopoiesis in response to alum (38).

The association of a cytokine profile characteristic of neutrophil recruitment and activation is in keeping with previous studies evaluating predictive biomarkers of mortality in children with sepsis. Both IL-1α and IL-1Ra are differentially expressed in survivors and nonsurvivors among children with sepsis (39) and IPD (11), respectively. However, raised plasma IL-8 in particular is well established as a predictor of mortality in children with sepsis. Serum IL-8 levels are increased at admission to pediatric intensive care units (PICU) in children with sepsis who subsequently die (40). In a prospective study, serum IL-8 of <220 pg/ml at admission to PICU had a 95% negative predictive value for mortality in children with sepsis (10).

The association of IL-8 with case fatality suggests that the determinants of mortality in iNTS disease considerably overlap those of undifferentiated sepsis. The data are also in keeping with observations that highlight a role for neutrophils in the control of invasive Salmonella infection. In African children, HIV infection and hemolysis (most notably secondary to malaria and hereditary hemolytic anemias) are important risk factors for iNTS disease (1). In both of these cases, enhanced susceptibility to iNTS disease appears in part to be secondary to impairment of neutrophil function, with HIV infection perturbing Th17-mediated recruitment of neutrophils to the intestinal mucosa (41) and hemolysis impairing neutrophil respiratory burst capacity (42).

A strength of this study is the recruitment of substantial numbers of children with sepsis secondary to a single pathogen, reflecting the importance of NTS as a cause of sepsis in African children. For future studies, it will be particularly important to address whether the cytokine signature predictive of mortality in iNTS disease is generalizable to sepsis secondary to other pathogens and to other populations. The use of multiplexed assays in this study facilitates an unbiased assessment of cytokine responses to iNTS disease but also imposes a burden of multiple-association testing with a consequent loss of study power. The limitations placed on study power by multiple-association testing may in part account for our failure to replicate well-validated correlates of mortality in sepsis (in particular, TNF [8], IL-6 [9], and IL-10 [6, 7]). Those discrepancies, however, may equally reflect differences in patient populations, causative pathogens, and sample collection timing. Larger follow-up studies could further define differences in cytokine responses in children with fatal disease and in groups of children with iNTS-associated comorbidities. Recruitment and blood sampling at admission (before a blood culture diagnosis of bacteremia is possible) is required to understand whether children who die early in admission with iNTS disease have the same cytokine profile as those who die later in admission.

In conclusion, circulating cytokine profiles of Malawian children with acute iNTS disease, regardless of HIV coinfection and nutritional status, are characterized by two coregulated groups of cytokines which represent distinct immunological responses. The first group is associated with the migration and activation of macrophages and may reflect the replicative niche of Salmonella within the macrophage. The second is characterized by concomitant pro- and anti-inflammatory immune responses typical of sepsis regardless of the causative pathogen. Children with fatal iNTS disease have distinctive cytokine profiles during acute disease, characterized by a combination of dysregulated IL-1 signaling, increased HGF levels, and increased IL-8 production. This signature may well result in the perturbation of the infiltration and activation of neutrophils in infected and damaged tissues and is associated with increased circulating neutrophil numbers. These findings are in keeping with our understanding of the biology of sepsis in children and with an increasing appreciation of the potential role of neutrophil recruitment (41) and function (42) in the control of Salmonella disease.

## Supplementary Material

Supplemental material
