# Supplementary material for: Cytokine Profiles during Invasive Nontyphoidal Salmonella Disease Predict Outcome in African Children
Source: Clin Vaccine Immunol. 2016 Jul 5;23(7):601–9. doi: 10.1128/CVI.00128-16 (PMC4933780; doi:10.1128/CVI.00128-16)
Supplement: Supplemental material [file supp_23_7_601__index.html]

Supplemental material 

# Cytokine Profiles during Invasive Nontyphoidal Salmonella Disease Predict Outcome in African Children

## Supplemental material

- Supplemental file 1 -

  Table S1. Cytokines, chemokines, and growth factors included in the analysis. Table S2. Serum cytokine concentrations during fatal and nonfatal iNTS disease. Table S3. Logistic regression model of iNTS disease mortality including principal components of acute serum cytokine concentrations. Table S4. Logistic regression model of iNTS disease mortality including the mortality-associated principal component of cytokine concentrations and NTS-associated comorbidities. Table S5. Linear regression model of peripheral blood neutrophil counts in acute iNTS disease.

  PDF, 427K
