# Supplementary material for: Cytokine Profiles during Invasive Nontyphoidal Salmonella Disease Predict Outcome in African Children
Source: Clin Vaccine Immunol. 2016 Jul 5;23(7):601–9. doi: 10.1128/CVI.00128-16 (PMC4933780; doi:10.1128/CVI.00128-16)
Supplement: Supplemental material [file CVI.00128-16_zcd999095370so1.pdf]

Table S1. Cytokines, chemokines and growth factors included in the analysis

Cytokine	Abbreviation	Minimum detected cytokine concentration (pg/ml)
Interferon-α2	IFNα2	8.43
Interleukin-3	IL-3	13.68
Leukemia inhibitory factor	LIF	1.87
Chemokine (C-C motif) ligand 7	CCL7	3.19
Interleukin-18	IL-18	64.33
KIT ligand	KITLG	32.38
Chemokine (C-X-C motif) ligand 12	CXCL12	30.62
Interleukin-1α	IL-1α	0.01
Hepatocyte growth factor	HGF	220.85
Colony stimulating factor-1	CSF1	0.06
Interleukin-1 receptor antagonist	IL-1Ra	52.29
Interleukin-8	IL-8	1.09
Chemokine (C-X-C motif) ligand 10	CXCL10	787.06
Nerve growth factor	NGF	1.41
Chemokine (C-C motif) ligand 27	CCL27	255.11
Interleukin 2 receptor a	IL-2Ra	101.37
Interleukin-12p40	IL-12p40	43.34
Interleukin-6	IL-6	3.92
Lymphotoxin a	LTA	0.07
Chemokine (C-X-C motif) ligand 9	CXCL9	25.19
Interleukin-10	IL-10	1.6
Interleukin-5	IL-5	0.12
C-type lectin domain family 11 member A	CLEC11A	10,961.07
Interleukin-15	IL-15	1.3
Colony stimulating factor 3	CSF3	1.28
Platelet-derived growth factor β polypeptide	PDGFβ	14.04
Chemokine (C-C motif) ligand 4	CCL4	34.65
Chemokine (C-C motif) ligand 2	CCL2	0.15
Interleukin-9	IL-9	0.06
Macrophage migration inhibitory factor	MIF	89.27
Chemokine (C-X-C motif) ligand 1	CXCL1	57.92
Interleukin-2	IL-2	0.66
Interferon-γ	IFNγ	9.66
Tumor necrosis factor (ligand) superfamily member 10	TNFSF10	58.59
Chemokine (C-C motif) ligand 11	CCL11	16.01
Chemokine (C-C motif) ligand 5	CCL5	292.83
Interleukin-16	IL-16	56.06
Interleukin-1β	IL-1β	0.23
Interleukin-4	IL-4	0.31
Interleukin-7	IL-7	0.76

Interleukin-12	IL-12	2.45
Interleukin-13	IL-13	0.6
Interleukin-17	IL-17	3.25
Fibroblast growth factor 2	FGF2	0.7
Colony stimulating factor 2	CSF2	0.3
Tumor necrosis factor	TNF	0.06
Vascular endothelial growth factor A	VEGFA	1.34
Chemokine (C-C motif) ligand 3	CCL3	1.61

Table S2. Serum cytokine concentrations during fatal and nonfatal iNTS disease

	Median (IQR) cytokine concentration (pg/ml)			
	Survived	Died	$P_{unadjusted}$	$P_{adjusted}$
**HGF**	**1,659 (1,035-2,697)**	**5,390 (2,801-7,115)**	**$1.3x10^{-5}$**	**$6.4x10^{-4}$**
**IL-8**	**34.1 (26.1-57.9)**	**131.3 (66.4-206.2)**	**$7.3x10^{-5}$**	**$3.4x10^{-3}$**
**IL-1Ra**	**697.7 (251.7-1,457.6)**	**5,339.9 (1,051.7-9,819.8)**	**$2.0x10^{-4}$**	**$9.2x10^{-3}$**
**IL-18**	**1,097 (616-1,795)**	**2,684 (1,967-4,948)**	**$2.2x10^{-4}$**	**0.01**
**IL-3**	**299.4 (166.2-437.1)**	**537.9 (411.1-716.1)**	**$3.4x10^{-4}$**	**0.02**
**IL-1α**	**2.4 (1.4-3.3)**	**4.0 (3.3-4.5)**	**$3.6x10^{-4}$**	**0.02**
**LIF**	**28.8 (4.2-54.4)**	**72.3 (52.2-94.9)**	**$3.8x10^{-4}$**	**0.02**
**IFNα2**	**154.6 (119.4-188.6)**	**220.3 (205.2-232.9)**	**$4.1x10^{-4}$**	**0.02**
**CXCL12**	**251.1 (184.9-305.4)**	**408.8 (283.4-540.6)**	**$4.5x10^{-4}$**	**0.02**
**CCL7**	**45.9 (28.6-63.6)**	**89.6 (72.2-93.4)**	**$8.0x10^{-4}$**	**0.03**
**KITLG**	**176.7 (120.8-250.8)**	**322.5 (233.7-387.2)**	**$8.3x10^{-4}$**	**0.03**
**CSF1**	**38.1 (27.2-60.7)**	**104.0 (48.1-158.0)**	**$1.1x10^{-3}$**	**0.04**
LTA	5.5 (3.4-7.7)	8.4 (6.9-11.4)	$1.8x10^{-3}$	0.07
CCL27	1,072 (826-1,391)	1,680 (1,290-1,914)	$2.1x10^{-3}$	0.07
NGF	9.0 (6.6-13.5)	15.8 (12.0-18.5)	$2.6x10^{-3}$	0.09
IL-16	264.0 (182.3-359.2)	402.1 (294.8-574.3)	$2.6x10^{-3}$	0.09
TNFSF10	371.1 (254.4-492.6)	555.9 (440.5-626.9)	$3.8x10^{-3}$	0.12
CCL4	230.8 (127.4-317.3)	340.9 (261.8-626.9)	$5.2x10^{-3}$	0.16
PDGFβ	4,131.3 (2,350.7-8,176.6)	864.7 (328.6-3,933.6)	0.01	0.31
IL-12p40	649.8 (444.6-957.2)	956.9 (626.9-1,313.4)	0.02	0.45
IL-6	38.9 (20.4-91.6)	103.1 (41.2-264.1)	0.02	0.51
CCL5	8,515 (6,098-11,082)	4,390 (1,803-10,793)	0.02	0.59
IL-2Ra	916 (539-1,453)	1,236 (1,025-3,069)	0.03	0.68
CCL2	14.8 (5.6-30.4)	32.6 (16.7-62.3)	0.03	0.70
IL-15	13.0 (1.7-23.9)	24.5 (13.4-31.8)	0.03	0.79
MIF	727.9 (464.7-1,128.7)	1,310.7 (641.9-2,126.7)	0.04	0.83

CCL11	166.5 (97.9-252.4)	247.3 (150.2-494.4)	0.04	0.85
CSF3	41.8 (22.9-63.4)	53.4 (35.2-399.5)	0.10	1.00
CXCL1	376.5 (241.1-641.7)	294.0 (134.2-436.8)	0.14	1.00
IL-5	0.8 (0.1-2.0)	0.1 (0.1-0.9)	0.15	1.00
CCL3	5.5 (1.6-11.3)	10.6 (2.4-16.1)	0.16	1.00
IL-10	14.5 (7.2-32.5)	18.7 (9.8-59.6)	0.16	1.00
CXCL9	11,656 (6,518-23,796)	18,431 (9,093-31,361)	0.24	1.00
IL-17	72.0 (38.2-143.7)	92.0 (39.7-217.4)	0.25	1.00
CXCL10	16,445 (9,223-29,322)	22,891 (17,663-41,272)	0.28	1.00
VEGFa	102.6 (52.8-150.3)	73.2 (13.7-148.2)	0.33	1.00
FGF2	23.5 (10.0-42.8)	16.4 (9.2-38.7)	0.39	1.00
CSF2	12.9 (0.3-25.1)	14.7 (5.1-49.5)	0.43	1.00
IL-12p70	39.2 (24.4-61.3)	38.9 (14.1-55.7)	0.46	1.00
TNF	5.5 (0.1-26.4)	2.7 (0.1-17.6)	0.57	1.00
IFNγ	393.1 (247.7-644.3)	325.1 (233.2-717.5)	0.67	1.00
IL-9	30.8 (15.9-51.9)	21.8 (16.3-64.2)	0.86	1.00
CLEC11a	61,008 (42,222-108,227)	74,930 (42,128-100,094)	0.86	1.00
IL-4	2.5 (1.5-3.7)	2.1 (1.5-3.1)	0.89	1.00
IL-7	6.1 (3.8-9.1)	6.6 (3.5-9.2)	0.90	1.00
IL-1β	1.8 (0.4-3.3)	1.4 (0.2-4.4)	0.92	1.00
IL-13	6.9 (2.1-12.3)	8.4 (1.9-12.1)	0.93	1.00
IL-2	11.6 (5.9-20.8)	11.0 (3.7-33.7)	0.98	1.00

Data from 108 children (14 died, 94 survived) are included in the analysis. Significance testing is with Mann-Whitney U tests, with *P* values adjusted for multiple comparisons with Holm step-down corrections. Serum cytokines concentrations significantly altered in fatal cases of iNTS disease ($P_{adjusted}<0.05$) are highlighted in bold. IQR, interquartile range.

Table S3. Logistic regression model of iNTS disease mortality including principal components of acute serum cytokine concentrations

	Adjusted Odds Ratio (95% confidence interval) for mortality	*P*
$PC1_{mortality}$	1.53 (0.44-6.33)	0.459
**$PC2_{mortality}$**	**4.04 (1.40-15.10)**	**0.004**

Data from 108 children (14 died, 94 survived) are included in the model. PC, principal component.

Table S4. Logistic regression model of iNTS disease mortality including the mortality-associated principal component of cytokine concentrations and NTS-associated comorbidities

	Adjusted Odds Ratio (95% confidence interval) for mortality	*P*
**$PC2_{mortality}$**	**5.11 (2.29-14.18)**	**3.6×10^{-4}**
HIV	2.14 (0.48-10.51)	0.320
Malnutrition	2.04 (0.34-11.25)	0.411

Data from 97 children (14 died, 83 survived) are included in the model. PC, principal component.

Table S5. Linear regression model of peripheral blood neutrophil counts in acute iNTS disease

	β coefficient (95% CI)	*P*
**$PC2_{mortality}$**	**0.24 (0.02-0.47)**	**0.04**
Age	0.00 (-0.01-0.01)	0.66
Sex	0.38 (-0.06-0.81)	0.10
Severe malnutrition	0.13 (-0.38-0.65)	0.62
HIV infection	0.11 (-0.32-0.53)	0.63

Data from 52 children are included in the model. PC, principal component.
